# Supplementary material for: An open-source framework for large-scale, flexible evaluation of biomedical text mining systems
Source: J Biomed Discov Collab. 2008 Jan 29;3:1. doi: 10.1186/1747-5333-3-1 (PMC2276192; doi:10.1186/1747-5333-3-1)
Supplement: Additional file 1 — Evaluation platform source code. The additional file contains the software evaluation framework discussed in this paper. The most current release can be downloaded from SourceForge.net [1]. See the accompanying README file for instructions on installation and use. Also included in the distribution is a collection of UIMA wrappers for some commonly used BioNLP tools and annotated corpora. [file 1747-5333-3-1-S1.zip › bionlp_uima_repository-1.0.1/docs/api/src-html/edu/uchsc/ccp/util/external/stanfordparser/TypedDependencyCls_Util.html]

```
001    package edu.uchsc.ccp.util.external.stanfordparser;
002    
003    import java.util.regex.Matcher;
004    import java.util.regex.Pattern;
005    
006    public class TypedDependencyCls_Util {
007    
008        /**
009         * parses a string taking the form of "gov:# dep:# rel:relation" and returns a TypedDependency
010         * 
011         * @return
012         */
013    public static TypedDependencyCls parseTypedDependencyClsString(String tdStr) throws InvalidTypedDependencyException {
014        int govToken = -1;
015        int depToken = -1;
016        String relation = "";
017        
018        Pattern govPattern = Pattern.compile("gov:([\\d]+)");
019        Matcher matcher = govPattern.matcher(tdStr);
020        
021        if (matcher.find()) {
022            govToken = Integer.parseInt(matcher.group(1));
023        } else {
024            System.err.println("Invalid Typed Dependency String. Missing governor token.");
025            throw new InvalidTypedDependencyException();
026        }
027        
028        Pattern depPattern = Pattern.compile("dep:([\\d]+)");
029        matcher = depPattern.matcher(tdStr);
030        
031        if (matcher.find()) {
032            depToken = Integer.parseInt(matcher.group(1));
033        } else {
034            System.err.println("Invalid Typed Dependency String. Missing dependent token.");
035            throw new InvalidTypedDependencyException();
036        }
037        
038        Pattern relPattern = Pattern.compile("rel:([\\w]+)");
039        matcher = relPattern.matcher(tdStr);
040        
041        if (matcher.find()) {
042            relation = matcher.group(1);
043        } else {
044            System.err.println("Invalid Typed Dependency String. Missing relation.");
045            throw new InvalidTypedDependencyException();
046        }
047        
048        return new TypedDependencyCls(govToken, depToken, relation);
049    }
050    
051    }
```
